# Sarcopenia is associated with mortality in non-critical elderly patients visiting the emergency department

**DOI:** 10.3389/fmed.2022.1027503

**Published:** 2023-01-11

**Authors:** Mei-Chen Liao, Cheng-Chang Yen, Yuh-Te Lin, Fong-Dee Huang, Yun-Te Chang

**Affiliations:** ^1^Center for Geriatrics and Gerontology, Kaohsiung Veterans General Hospital, Kaohsiung City, Taiwan; ^2^Division of Neurology, Department of Internal Medicine, Kaohsiung Veterans General Hospital, Kaohsiung City, Taiwan; ^3^Department of Emergency Medicine, Kaohsiung Veterans General Hospital, Kaohsiung City, Taiwan; ^4^School of Medicine, National Yang-Ming University, Taipei City, Taiwan; ^5^Department of Physical Therapy, Shu-Zen Junior College of Medicine and Management, Kaohsiung City, Taiwan; ^6^Department of Emergency & Critical Care Medicine, Pingtung Veterans General Hospital, Pingtung City, Taiwan

**Keywords:** sarcopenia, geriatric syndrome, systemic inflammation, elderly emergency, prognosis

## Abstract

**Introduction:**

Geriatric syndrome (GS) increases risk of disability and mortality in older adults. Sarcopenia is a predominant illness of GS and accelerate its progression. This study aimed to investigate associations between mortality, emergency department (ED) re-visits and GS-related illnesses among older adults who visited the ED.

**Method:**

This retrospective observational study enrolled elderly patients who visited the ED in our hospital between January 2018 and October 2020. Patients were evaluated for potential sarcopenia, which was defined by both low handgrip strength and calf circumference. Follow-up was at least 6 months. Data of age, gender, mortality, ED re-visits, and GS-related illnesses were collected and analyzed for associations.

**Results:**

A total of 273 older adults aged 74 years or older were included, of whom 194 were diagnosed with possible sarcopenia. Older adults with possible sarcopenia also had significantly lower body mass index (BMI); a higher proportion needed assistance with daily activities; more had malnutrition, frailty, and history of falls (all *p* < 0.001) and acute decline in activities of daily living (*p* = 0.027). Multivariate analysis showed that possible sarcopenia [adjusted hazard ratio, aHR): 9.89, 95% confidence interval (CI): 1.17–83.81, *p* = 0.036], living in residential institutions (aHR: 2.85, 95% CI: 1.08–7.50, *p* = 0.034), and frailty (aHR: 7.30, 95% CI: 1.20–44.62, *p* = 0.031) were associated with mortality. Aged over 85 years (adjusted odds ratio: 2.44, 95% CI: 1.25–4.80, *p* = 0.02) was associated with ED re-visits.

**Conclusion:**

Sarcopenia is associated with mortality among older adults who visit ED. Initial screening for sarcopenia and relevant risk factors among older adults in the ED may help with early intervention for those at high-risk and may improve their prognosis.

## Introduction

Geriatric syndromes (GS) are multifactorial illnesses prevalent in geriatric populations (1, 2). Patients with GS may present with various physiological or psychiatric illnesses, including delirium, dementia, cognitive impairment, urinary incontinence, frailty, and mobility impairment (3). These disorders reduce the mobility of patients with GS, obstruct their connection to communities they belong, and subsequently increase their risk of disability and cognitive impairment (4, 5). Furthermore, those with GS need additional treatments frequently, including medications, nursing, hospitalization, or residential care, and may limit budgets and government funds for people of other ages in individual households (6–8). Therefore, monitoring the dynamic trends of GS may assist healthcare administrators or government policy-makers to implement policies to reduce the disadvantages of GS-associated illnesses among older adults.

To understand the dynamic of GS in a given population, it is necessary to comprehensively investigate the incidence of GS-associated illnesses and analyze their crosstalk. However, the progression of GS is multifactorial, and the interaction between GS-associated illnesses is complicated (1). Nevertheless, several pivotal factors are identified that contribute to GS, such as malnutrition, sarcopenia, and chronic diseases (9, 10). Sarcopenia describes a chronic loss of muscle mass and function for which diagnostic criteria include handgrip strength lower than 28 kg in men and 16 kg in women, calf circumference less than 34 cm in men and 33 cm in women, and 6-m walking speed less than 0.8 m per second in both genders(11). Sarcopenia obstructs mobility and impairs the balance of older adults, which correlates directly with the incidence of social isolation, frailty, and disability in the geriatric population (12, 13). Of note, sarcopenia involves the progression of GS but the level of involvement varies between ethnic groups and countries (14–16). Previous studies showed that 9% elders in Yilan county, Taiwan had sarcopenia (17) and the prevalence of sarcopenia was about 10% in the world (18). Because of the aging population, the dynamics of GS are monitored consistently in Taiwan (19). However, the association between GS and age-related clinical outcomes is still unclear. This study aimed to investigate the associations between mortality, ED re-visits, level of cytokines, and GS-related illnesses among older adults who visited the ED.

## Patients and methods

### Study design and population

In this retrospective observational study, elderly patients who visited the ED of our hospital between January 2018 and October 2020 were enrolled. Inclusion criteria were: (1) over the age of 74 years; (2) with follow-up for at least 6 months. Those with missing information on grip strength or calf circumference were excluded.

The European and Asian Sarcopenia Working Groups have proposed that muscle mass, muscle strength, and physical performance are three indicators used for the evaluation of sarcopenia (20, 21). Therefore, diagnosis of sarcopenia in this study was based on the following three procedures: muscle strength by handgrip strength lower than or equal to 28 kg in males or 16 kg in females, calf circumference less than 34 cm in males or 33 cm in females, and 6-m walking speed < 0.8 m/s in both males and females, as previously described (22, 23). We defined subjects diagnosed with sarcopenia and severe sarcopenia as “possible” sarcopenia. Other subjects were defined as subjects without sarcopenia (non-sarcopenia).

### Outcomes

The primary outcomes of interest were mortality and re-visits to the ED within 6 months during follow-up.

### Evaluating geriatric syndrome

Grading of frailty, depression, malnutrition, and Charlson’s comorbidity index (CCI) were used for analysis in this study. Depression was graded by a five-item Geriatric Depression Scale (24). Participants had grade ≥ 2 were considered as depression. Mini-Nutrition assessment-short form was used for evaluating malnutrition (25). Participants had score lower than 12 were considered as malnutrition. Frailty score was determined by the Cardiovascular Health Study (26). CCI was evaluated following Age-adjusted Charlson’s comorbidity index (27).

### Monitoring cytokines

Blood was collected into a BD Vacutainer™ EDTA Blood Collection Tubes (8 mL) and kept on ice until processing. The blood samples were centrifugated at 3,000 rpm for 10 min and the supernatants were frozen in –20°C until analysis.

Human TNF-α ELISA kit (ARG80120, Arigo biolaboratories, Hsinchu city, Taiwan) and Human IL-6 ELISA kit (ARG80110, Arigo biolaboratories) were used to analyze the concentration of TNF-α and IL-6 in the serum, respectively. The procedures suggested by the manufacturer was followed to determine the cytokine concentrations.

### Statistical analysis

The Shapiro–Wilk test was used for continuous data with non-normal distribution, including grip strength, calf circumference, and BMI, which are presented as medians (interquartile: 25–75th percentile, IQR). Categorical variables were estimated as frequency and percentage, including gender, age, education level, marital status, living status, daily activities, Charlson comorbidity index (CCI), ED discharge destination, and GS components. Hazard ratios (HRs) were estimated using the Cox proportional-hazard model to present the effects of covariates on mortality. Logistic regression analysis was used to explore associations between covariates and ED re-visits. Odds ratios (ORs) were used to present the associations between high cytokine levels, the presence of possible sarcopenia at ED, and mortality based on the logistic regression model. All statistics were two-sided and all statistical analysis was performed using SAS statistical software (version 9.4, SAS, Cary, NC, USA). A *P*-value < 0.05 was established as statistical significance.

## Results

### Baseline characteristics of the study population

**FIGURE 1:**
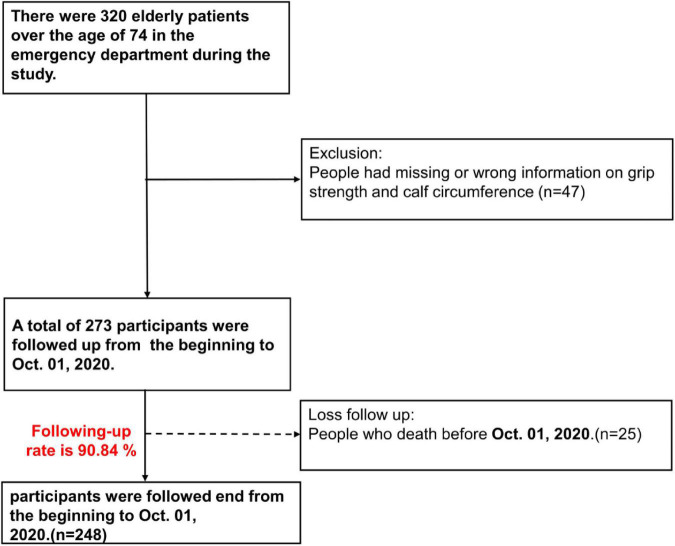
Flow diagram of study selection.

In total, 320 patients were enrolled in this study. After excluding, 273 patients were including in the cohort. Unfortunately, 25 patients were lost of follow-up due to death in the study period and the follow-up rate was 90.84% (248/273) (Figure 1). Among the subjects, 194 patients were identified as possible sarcopenias. The prevalence of possible sarcopenia in our study population is 71.06%. Table 1 demonstrates the demographic and anthropometric characteristics of the study population as well as clinical signs of GS. Significant differences were found between possible sarcopenia and non-sarcopenia groups, including lower median grip strength [males: 17.3 (13.50–21.10) vs. 26.2 (19.40–26.20), females: 10.0 (7.90–12.80) vs. 16.50 (13.00–16.50)] and calf circumference [males: 30.0 (27.5–32.0) vs. 35.0 (34.0–36.50), females: 28.5 (26.5–31.0) vs. 33.00 (30.25–34.25)] (all *p* < 0.001). The proportion of possible sarcopenia was comparable in both genders (males 70.9% vs. females 71.22%, *p* = 0.952). Subjects with possible sarcopenia had significantly lower BMI [23.19 (20.96–25.39) vs. 26.79 (24.13–29.76), *p* < 0.001]; higher percentages of those needing assistance with daily activities (80.95% vs. 19.05%; *p* < 0.001), and acute ADL decline (72.98% vs. 27.02%, *p* = 0.027); malnutrition (MNASF < 12) (79.33% vs. 20.67%, *p* < 0.001), frailty (Cardiovascular Health Study ≥ 3) (84.88% vs. 15.12%, *p* < 0.001) (26), and history of falls (85.42% vs. 14.58%, *p* < 0.001).

**TABLE 1:** Baseline characteristics of elderly patients at ED (*n* = 273).

Variables	Possible sarcopenia		*P*-value
	Yes (*n* = 194)	No (*n* = 79)	
**Demographic and anthropometric data**			
Grip strength (kg)			
Male	17.3 (13.50–21.10)	26.2 (19.40–26.20)	**<0.001**
Female	10.0 (7.90–12.80)	16.50 (13.00–16.50)	**<0.001**
Calf circumference (cm)			
Male	30.0 (27.5–32.0)	35.0 (34.0–36.5)	**<0.001**
Female	28.5 (26.5–31.0)	33.00 (30.25–34.25)	**<0.001**
Gender			0.952
Male	95 (70.90)	39 (29.10)	
Female	99 (71.22)	40 (28.78)	
Age (years)			**0.020**
75–79	52 (62.65)	31 (37.35)	
80–84	65 (68.42)	30 (31.58)	
85–100	77 (81.05)	18 (18.95)	
BMI	23.19 (20.96–25.39)	26.79 (24.13–29.76)	**<0.001**
Education level			0.072
Low	79 (77.45)	23 (22.55)	
High	115 (67.25)	56 (32.75)	
Marital status			0.370
Without spouse	100 (73.53)	36 (26.47)	
With spouse	94 (68.61)	43 (31.39)	
Living status			0.407
Live at home	170 (70.25)	72 (29.75)	
Residential institutions	24 (77.42)	7 (22.58)	
Daily activities			**<0.001**
Independent	75 (59.52)	51 (40.48)	
Need assistance	119 (80.95)	28 (19.05)	
Charlson comorbidity index			0.334
0–1	131 (68.59)	60 (31.41)	
2	35 (74.47)	12 (25.53)	
≥3	28 (80.00)	7 (20.00)	
ED discharge destination		0.992	
Hospitalization	145 (71.08)	59 (28.92)	
Discharge	49 (71.01)	20 (28.99)	
**Components of geriatric syndrome**			
Acute ADL decline			
Yes	181 (72.98)	67 (27.02)	**0.027**
No	13 (52.00)	12 (48.00)	
Malnutrition (MNASF < 12)			**<0.001**
Yes	142 (79.33)	37 (20.67)	
No	52 (55.32)	42 (44.68)	
Frailty (CHS ≥ 3)			**<0.001**
Yes	146 (84.88)	26 (15.12)	
No	48 (47.52)	53 (52.48)	
Multiple medications^a^			0.062
Yes	115 (76.16)	36 (23.84)	
No	51 (64.56)	28 (23.84)	
Poor eyesight/hearing			0.262
Yes	98 (74.24)	34 (25.76)	
No	96 (68.09)	45 (31.91)	
Sleep problems			0.665
Yes	100 (69.93)	43 (30.07)	
No	94 (72.31)	36 (27.69)	
Cognitive impairment			0.091
Yes	67 (77.91)	19 (22.09)	
No	127 (67.91)	60 (32.09)	
Depression (GDS ≥ 2)			0.203
Yes	85 (75.22)	28 (24.78)	
No	109 (68.13)	51 (31.88)	
History of falls			**<0.001**
Yes	82 (85.42)	14 (14.58)	
No	112 (63.28)	65 (36.72)	
Defecation problems			0.487
Yes	39 (75.00)	13 (25.00)	
No	155 (70.14)	66 (29.86)	
Urinary incontinence			0.213
Yes	77 (75.49)	25 (24.51)	
No	117 (68.42)	54 (31.58)	
Pain			0.072
Yes	33 (61.11)	21 (38.89)	
No	161 (73.52)	58 (26.48)	
Mortality	24 (12.37%)	1 (1.27%)	0.004

ADL, activities of daily living; BMI, body mass index; CHS, cardiovascular health study; ED, emergency department; GDS, geriatric depression scale; IQR; interquartile range; MNA-SF, mini nutritional assessment-short form. Continuous data are presented as median (IQR) and categorical data are presented as *n* (%). Significance values are in bold. ^a^Missing for 43 patients.

### Study outcomes

Table 2 presents the associations between study variables and mortality. Univariate Cox PH model showed that mortality was associated with possible sarcopenia (crude HR = 9.75, 95% CI: 1.32–72.21, *p* = 0.026), living in residential institutions (crude HR = 3.17, 95% CI: 1.23–8.14, *p* = 0.017), frailty (crude HR = 5.88, 95% CI: 1.38–25.07, *p* = 0.017), cognitive impairment (crude HR = 3.14, 95% CI: 1.42–6.93, *p* = 0.005), and defecation problems (crude HR = 2.79, 95% CI: 1.26–6.19, *p* = 0.012).

**TABLE 2:** Associations between study variables and mortality (*n* = 273).

Variables	Mortality							
	Crude HR (95% CI) *P*-value		Model 1^a^		Model 2^b^		Model 3^c^	
			aHR (95% CI)	*P*-value	aHR (95% CI)	*P*-value	aHR (95% CI)	*P*-value
Possible sarcopenia (vs. no sarcopenia)	**9.75 (1.32–72.21)**	**0.026**	4.86 (0.63–37.68)	0.130	4.48 (0.49–41.35)	0.186	**9.89 (1.17–83.81)**	**0.036**
**Demographic and anthropometric data**								
**Gender**								
Male	Ref				Ref		Ref	
Female	0.61 (0.27–1.35)	0.221			**0.18 (0.05–0.59)**	**0.005**	**0.29 (0.10**–**0.89)**	**0.030**
**Age**								
75–79	Ref				Ref		Ref	
80–84	2.13 (0.73–6.28)	0.169			1.13 (0.30–4.17)	0.860	1.60 (0.44–5.88)	0.480
85–100	1.58 (0.54–4.65)	0.404			0.37 (0.09–1.65)	0.194	0.76 (0.20–2.91)	0.692
BMI	0.96 (0.87–1.06)	0.376			1.00 (0.96–1.04)	0.848	1.00 (0.97–1.02)	0.806
**Education level**								
Low	0.75 (0.32–1.74)	0.503			1.93 (0.38–9.86)	0.430	0.63 (0.22–1.80)	0.391
High	Ref				Ref		Ref	
**Marital status**								
Without spouse	Ref				Ref		Ref	
With spouse	0.98 (0.45–2.14)	0.955			0.60 (0.23–1.57)	0.301	0.62 (0.24–1.58)	0.315
**Living status**								
Live at home	Ref		Ref		Ref			
Residential institutions	**3.17 (1.23–8.14)**	**0.017**	**2.85 (1.08–7.50)**	**0.034**	2.50 (0.76–8.22)	0.132	–	
Daily activities								
Independent	Ref				Ref			
Need assistance	1.39 (0.61–3.15)	0.431			0.83 (0.30–2.31)	0.714	–	
**Charlson comorbidity index**								
0–1	Ref				Ref		Ref	
2	1.11 (0.37–3.30)	0.849			0.56 (0.14–2.33)	0.424	0.60 (0.16–2.32)	0.461
≥3	0.86 (0.20–3.75)	0.842			0.33 (0.06–1.88)	0.212	0.36 (0.07–1.96)	0.236
**ED discharge destination**								
Hospitalization	1.13 (0.45–2.84)	0.790			0.92 (0.32–2.61)	0.872	1.01 (0.35–2.92)	0.988
Discharge	Ref				Ref		Ref	
**Components of geriatric syndrome**								
Acute ADL decline	2.33 (0.31–17.33)	0.407			2.67 (0.27–26.41)	0.401	2.56 (0.29–22.48)	0.397
Malnutrition (MNA-SF < 12)	2.01 (0.75–5.38)	0.165			0.51 (0.15–1.72)	0.278	1.00 (0.31–3.19)	0.998
Frailty (CHS ≥ 3)	**5.88 (1.38–25.07)**	**0.017**	3.40 (0.76–15.18)	0.109	**7.30 (1.20–44.62)**	**0.031**	–	
Multiple medications^d^	2.24 (0.83–5.99)	0.110			2.38 (0.73–7.77)	0.152	2.07 (0.68–6.32)	0.202
Poor eyesight/hearing	0.90 (0.41–1.98)	0.796			0.79 (0.31–2.06)	0.635	0.79 (0.322–1.94)	0.607
Sleep problems	1.41 (0.63–3.14)	0.401			2.52 (0.86–7.36)	0.091	1.75 (0.69–4.48)	0.242
Cognitive impairment	**3.14 (1.42–6.93)**	**0.005**	2.19 (0.97–4.95)	0.059	3.46 (0.80–14.90)	0.096	–	
Depression (GDS ≥ 2)	0.89 (0.40–1.99)	0.782			0.37 (0.12–1.15)	0.086	0.61 (0.24–1.57)	0.307
History of falls	1.53 (0.70–3.36)	0.291			1.32 (0.52–3.35)	0.556	1.22 (0.49–3.07)	0.671
Defecation problems	**2.79 (1.26–6.19)**	**0.012**	1.81 (0.79–4.17)	0.161	1.12 (0.43–2.89)	0.823	1.50 (0.61–3.73)	0.378
Urinary incontinence	1.60 (0.73–3.51)	0.243			1.59 (0.60–4.20)	0.352	1.68 (0.66–4.27)	0.273
Pain	1.44 (0.57–3.62)	0.440			1.69 (0.46–6.26)	0.432	1.98 (0.57–6.92)	0.284

ADL, activities of daily living; BMI, body mass index; CHS, cardiovascular health study; ED, emergency department; GDS, geriatric depression scale; IQR, interquartile range; HR, hazard ratio; aHR, adjusted HR; CI, confidence interval; MNA-SF, mini nutritional assessment-short form. Significant values are presented in bold. ^a^Model 1: adjusted for variables (*p* < 0.10) in the univariate analysis. ^b^Model 2: adjusted for all variables. ^c^Model 3: adjusted for all variables except frailty, daily activity, living status, and cognitive impairment. ^d^230 patients were included in the analysis.

**FIGURE 2:**
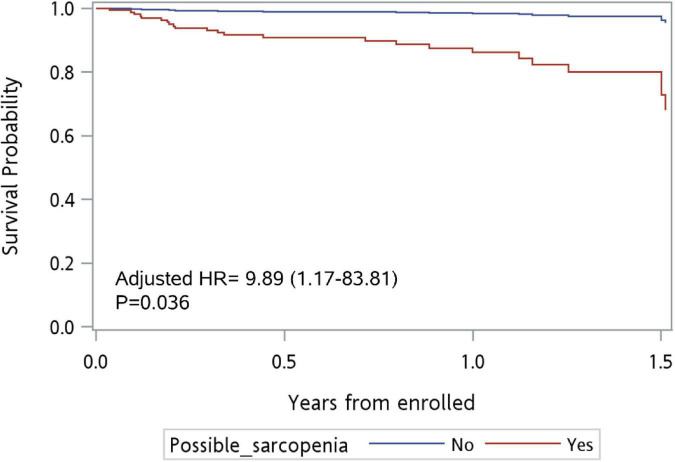
Survival curve for patients with possible sarcopenia vs. no sarcopenia at ED (*n* = 273). Adjusted for all variables except frailty, daily activity, living status, and cognitive impairment.

In the multivariate model, living in residential institutions [adjusted HR (aHR): 2.85, 95% CI: 1.08–7.50, *p* = 0.034] was an independent risk factor for mortality after adjusting for variables with *p*-value < 0.1, including possible sarcopenia, frailty, cognitive impairment, and defecation problems (Model 1). On the other hand, frailty (aHR: 7.30, 95% CI: 1.20–44.62, *p* = 0.031) was an independent risk factor for mortality after adjusting for all variables (Model 2). Possible sarcopenia (aHR = 9.89; 95% CI: 1.17–83.81, *p* = 0.036; Figure 2) was an independent risk factor for mortality after adjusting for all variables except frailty, daily activities, living status, and cognitive impairment (Model 3). Compared to males, females had lower risk for mortality (Model 2, aHR: 0.18, 95% CI: 0.05–0.59, *p* = 0.005; Model 3, aHR: 0.29, 95%CI: 0.1–0.89, *p* = 0.03).

Table 3 presents the associations between study variables and ED re-visits. Univariate logistic regression model showed ED re-visits were associated with age group 85–100 years (compared to 75–79 years group: crude OR = 2.56; 95% CI: 1.33–4.92, *p* = 0.02) and malnutrition (crude OR = 1.83; 95% CI: 1.05–3.18, *p* = 0.032). The multivariate model showed that age group 85–100 years was an independent risk factor for ED re-visits (adjusted OR: 2.44, 95% CI: 1.25–4.80, *p* = 0.02) after adjusting for possible sarcopenia, variables with *p*-value < 0.1, including marital status, malnutrition, and frailty (Model 1).

**TABLE 3:** Associations between study variables and ED re-visits (*n* = 273).

Variables	ED re-visit							
	Crude OR (95% CI)	*P*-value	Model 1^a^ aOR (95% CI)	*P*-value	Model 2^b^ aOR (95% CI)	*P*-value	Model 3^c^ aOR (95% CI)	*P*-value
Possible sarcopenia (vs. no sarcopenia)	1.17 (0.67–2.04)	0.590	0.77 (0.40–1.46)	0.415	0.46 (0.18–1.14)	0.093	0.69 (0.31–1.55)	0.366
**Demographic and anthropometric data**								
Gender								
Male	Ref				Ref		Ref	
Female	1.11 (0.68–1.84)	0.674			0.95 (0.43–2.12)	0.903	1.07 (0.49–2.32)	0.864
Age								
75–79	Ref		Ref		Ref		Ref	
80–84	1.79 (0.92–3.48)	0.675	1.66 (0.84–3.27)	0.836	0.94 (0.40–2.19)	0.419	1.01 (0.44–2.30)	0.431
85–100	**2.56 (1.33–4.92)**	**0.016**	**2.44 (1.25–4.80)**	**0.020**	1.53 (0.63–3.72)	0.211	1.72 (0.73–4.06)	0.127
BMI	0.95 (0.89–1.01)	0.125			0.93 (0.85–1.02)	0.106	0.94 (0.86–1.03)	0.176
Education level								
Low	1.54 (0.92–2.57)	0.100			2.22 (0.94–5.22)	0.069	1.93 (0.84–4.44)	0.121
High	Ref				Ref		Ref	
Marital status								
Without spouse	Ref		Ref		Ref		Ref	
With spouse	0.60 (0.36–1.00)	0.051	0.64 (0.38–1.09)	0.098	0.88 (0.43–1.82)	0.737	0.80 (0.40–1.60)	0.535
Living status								
Live at home	Ref				Ref			
Residential institutions	1.70 (0.80–3.62)	0.170			1.80 (0.68–4.78)	0.241	–	
Daily activities								
Independent	Ref				Ref			
Need assistance	1.21 (0.73–2.01)	0.454			0.84 (0.44–1.64)	0.617	–	
Charlson comorbidity index								
0–1	Ref				Ref		Ref	
2	0.57 (0.28–1.16)	0.121			0.45 (0.18–1.08)	0.073	0.48 (0.20–1.14)	0.097
≥3	0.57 (0.25–1.29)	0.178			0.44 (0.16–1.19)	0.105	0.45 (0.17–1.17)	0.101
ED discharge destination								
Hospitalization	0.63 (0.36–1.11)	0.108			0.61 (0.31–1.20)	0.149	0.65 (0.34–1.26)	0.203
Discharge	Ref				Ref		Ref	
**Components of geriatric syndrome**								
Acute ADL decline	2.20 (0.80–6.06)	0.128			1.96 (0.60–6.38)	0.266	1.94 (0.61–6.21)	0.264
Malnutrition (MNA-SF < 12)	**1.83 (1.05–3.18)**	**0.032**	1.51 (0.83–2.75)	0.177	1.58 (0.75–3.32)	0.230	1.76 (0.86–3.60)	0.125
Frailty (CHS ≥ 3)	1.71 (1.00–2.92)	0.051	1.49 (0.80–2.74)	0.206	2.53 (1.11–5.74)	0.027	–	
Multiple medications^d^	0.91 (0.52–1.60)	0.741			1.08 (0.54–2.17)	0.829	1.06 (0.53–2.11)	0.868
Poor eyesight/hearing	0.94 (0.57–1.55)	0.805			0.63 (0.33–1.20)	0.160	0.69 (0.37–1.28)	0.238
Sleep problems	1.33 (0.80–2.19)	0.274			1.51 (0.79–2.89)	0.209	1.51 (0.80–2.85)	0.207
Cognitive impairment	1.22 (0.72–2.09)	0.458			1.13 (0.49–2.62)	0.778	1.18 (0.52–2.69)	0.691
Depression (GDS ≥ 2)	0.97 (0.58–1.61)	0.898			0.55 (0.27–1.09)	0.087	0.70 (0.37–1.33)	0.274
History of falls	1.36 (0.81–2.28)	0.251			1.40 (0.71–2.79)	0.332	1.34 (0.69–2.62)	0.390
Defecation problems	1.27 (0.68–2.37)	0.458			0.90 (0.41–1.96)	0.791	0.99 (0.46–2.12)	0.977
Urinary incontinence	1.25 (0.75–2.09)	0.391			1.39 (0.73–2.63)	0.320	1.33 (0.71–2.48)	0.368
Pain	0.87 (0.46–1.64)	0.655			0.66 (0.28–1.54)	0.331	0.70 (0.30–1.62)	0.404

ADL, activities of daily living; BMI, body mass index; CHS, cardiovascular health study; ED, emergency department; GDS, geriatric depression scale; IQR, interquartile range; HR, hazard ratio; aHR, adjusted HR; CI, confidence interval; MNA-SF, mini nutritional assessment-short form. Significance values are presented in bold. ^a^Model 1: adjusted for variables (*p* < 0.10) in the univariate analysis. ^b^Model 2: adjusted for all variables. ^c^Model 3: adjusted for all variables except frailty, daily activity, and living status. ^d^230 patients were included in the analysis.

No associations were found between the level of interleukin-6 (IL-6) and tumor necrosis factor alpha (TNF-α) and the presence of possible sarcopenia in ED patients (Table 4). Nor did mortality associate with cytokine level or cytokine level plus possible sarcopenia (Table 5).

**TABLE 4:** Associations between high cytokine levels and presence of possible sarcopenia at ED (*n* = 68).

Variables	Possible sarcopenia	*P*-value
	Crude OR (95% CI)	
**Cytokines**		
IL-6, continuous	1.00 (1.00–1.01)	0.399
High IL-6 (>17 pg/mL)	1.36 (0.42–4.45)	0.607
TNF-α, continuous	1.00 (1.00–1.01)	0.492
High TNF-α (>12.4 pg/mL)	1.02 (0.34–3.04)	0.974
High in both^a^	1.09 (0.38–3.09)	0.876

OR, odds ratio; CI, confidence interval; IL, interleukin; TNF, tumor necrosis factor. ^a^IL-6 > 17 pg/mL or TNF-α > 12.4 pg/mL.

**TABLE 5:** Associations between high cytokine level, possible sarcopenia at ED, and mortality.

Variables			Mortality					
			Crude OR	*P*-value	aOR	*P*-value	aOR	*P*-value
	Total	Death	(95% CI)		(95% CI)^a^		(95% CI)^c^	
**Cytokine level (*n* = 68)**								
Low	**33**	**5 (15.15)**	Ref		Ref		Ref	
High^e^	**35**	**6 (17.14)**	1.16 (0.32–4.23)	0.824	>999.99 (<0.01–>999.99)^b^	0.176	4.73 (0.36–62.29)	**0.238**
**Cytokine level in patients with possible sarcopenia (*n* = 48)**								
Low	**23**	**5 (21.74)**	Ref		Ref		Ref	
High^e^	**25**	**6 (24.00)**	1.14 (0.30–4.39)	0.852	>999.99 (<0.01–>999.99)^d^	0.902	7.76 (0.38–159.11)	**0.184**

OR, odds ratio; aOR, adjusted OR; CI, confidence interval. Significant values are in bold. ^a^Adjusted for all variables except frailty, daily activity, living status, and cognitive impairment. ^b^60 patients were included in the analysis. ^c^Adjusted for all variables except frailty, daily activity, living status, multiple medications, and cognitive impairment. ^d^42 patients were included in the analysis. ^e^IL-6 > 17 pg/mL or TNF-α > 12.4 pg/mL.

## Discussion

The present study found that older adults with sarcopenia also had lower BMI, higher percentages of acute ADL decline, malnutrition, frailty, and history of falls. Among these older adults who visited the ED, mortality was associated with gender, possible sarcopenia, living in residential institutions, and frailty. ED re-visits were associated with age older than 85 years. Of note, elderly ED patients with sarcopenia did not exhibit higher concentrations of IL-6 and TNF-α, which indicated that possible sarcopenia did not correlate with systemic inflammation.

Xu et al. reported that sarcopenia was associated with 3-month and 1-year mortality in geriatric rehabilitation inpatients (28). They also did a meta-analysis and reported that sarcopenia was associated with significantly higher risks of mortality in adults independent of population (29). Although the target subjects in our study are not specified in their comparison, our conclusion can be strengthened by their analysis. It is apparent that sarcopenia affects mortality in non-critical elderly patients visiting the emergency department.

In the present study, possible sarcopenia, frailty, and living in a residential institution contributed to increased geriatric mortality. Sarcopenia and frailty are known to elevate the risk of hospitalization among older adults (30, 31). Appetite decline causes malnutrition and frailty in older adults and further exacerbates sarcopenia and osteoporosis due to the decline in daily activities (12, 32–34). Furthermore, the decline in daily activities is also linked to cognitive impairment and increased mortality in the geriatric population (35, 36). Therefore, the positive relationship between sarcopenia, frailty, and risk of hospitalization among older adults is foreseeable. Interestingly, nursing home or long-term care residents exhibited a higher risk of mortality than those who lived at home. Previous studies revealed that nursing home residents do not need to take care of their daily life so they spend more time doing nothing or watching TV than those living at home or a private dwelling (37–39). This suggests that the negative impact on the health status of older adults living in residential institutions may be the result of the decline in daily activities.

Multivariate regression analysis also revealed that mortality in men was significantly higher than that in women. Gender difference have been observed as well in life expectancy (40). However, the pivotal factors causing gender differences vary between different countries (40). Elderly men in China have a higher willingness to utilize long-term care institutions than women (41). Also, frailty is more prevalent among elderly men in China than among women (42). Bellettiere et al. and Chen et al. both reported that elderly men had fewer daily activities than women; the demand for assistance with daily activities was also stronger in men than that in women (43, 44. These studies revealed that the potential cause of gender differences in mortality in China may be associated with the frailty that results from reduced daily activities in long-term care institutions. However, two investigations of older adults in Spain and Italy showed that elderly women had a higher risk of frailty and disability than men due to differences in mechanical and psychological patterns (45, 46). Despite that the life expectancy of women in these two populations is higher than that of men (47), mechanical and psychological patterns and disability do not seem to be the critical factors in gender differences in life expectancy. The present study preliminarily exhibited lower mortality in elderly women than that in elderly men but further studies are needed to identify the underlying factors.

Age older than 85 years was an independent risk factor for ED re-visits in the present study. This is the first study to report a correlation between age and frequency of ED revisits. An observational study in Turkey reported that the ED-visiting rate, or frequency, was negatively associated with age and elders aged between 65 and 74 years constituted the ED-visiting population with the most frequent visits, largely due to upper respiratory tract infections and chest pain (48). A similar trend was also observed in clinical investigations in America and Australia (49, 50). Of note, an observational trial in Finland revealed a positive correlation between the frequency of ED-visiting and the age of elders (51). The predominant diseases that cause older adults to visit the ED frequently are cardiovascular diseases such as heart failure and atrial fibrillation (51). Although the correlation between age and frequency of ED visits differs between countries, the complaints that contribute to ED visits are universal. In the present investigation, we discovered a positive correlation between age and ED re-visits, but the predominant complaint was unclear. Further investigations are needed to evaluate the causes of frequent visits to the ED, and results may help hospital and government policy-makers develop appropriate policies to address the complaints and thereby reduce ED visits.

Surprisingly, older adults with possible sarcopenia did not concurrently exhibit a higher level of IL-6 and TNF-α than non-sarcopenia subjects in our study. IL-6 and TNF-α are both inflammatory cytokines. Previous studies had shown that inflammatory cytokines were involved with muscle wasting and high level of those cytokines were negatively related to muscle strength and mass (52–56). Bian et al. also reported that the emergence of sarcopenia was accompanied by increased levels of TNF-α and IL-6 in elderly (57). Our study had completely opposite conclusion as their reports. In the study of Bian et al. (57) elders aged more than 60 years old and can walk by themselves or stand for 5 min with other auxiliaries were included. The percentage of subjects identified as sarcopenia was 17.9% (79/441). On the contrary, our subjects were recruited when they were admitted in the emergency department and they were older than 75 years old and the ratio of sarcopenia was 71.06%. Thus, subjects in our study were older, weaker, and having less muscle mess and strength.

In recent knowledge, pivotal links between sarcopenia and inflammatory cytokines include gut dysbiosis, malnutrition, and reduced daily activities. Gut dysbiosis, the imbalance of gut microbiota, affects nutrition absorption in the entericus and causes malnutrition to some extent (58, 59). Increased daily activities or physical exercises improve the appetite, which may reduce the risk of sarcopenia (60, 61). Moreover, exercises can trigger gut microbiota secreting short-chain fatty acids with anti-inflammatory effects, reduce oxidative stress, and maintain muscle mass (62–64). Older adults having pro-inflammatory diets may potentiate the incident risk of sarcopenia (65). More extensive study is needed to clarify the underlying mechanisms.

### Limitations

This study has a few limitations, mainly lying in the inherent restrictions of analyzing retrospective data, including that additional follow-up data were not available. Results of the single-center study in Taiwan also cannot be easily generalized to other populations or locations. However, observational studies focusing on a narrow topic may help to reduce the inherent limitations to a minimum. Results of the appendicular skeletal muscle mass index (direct evidence of sarcopenia) were missing for all patients, which would have fully demonstrated sarcopenia (66), providing a more accurate measure of possible sarcopenia in ED patients. Besides, cytokine levels were evaluated only in the minority of patients. This let it is impossible to apply the results to general geriatric population.

## Conclusion

Sarcopenia is highly associated physical condition, like BMI, frailty, and living in residential institutions, rather than systemic inflammation in the elders. And the mortality of ED-visiting elders is associated with gender and possible sarcopenia.

Findings of the present study may serve as a reference to guide ED management and to develop an appropriate ED examination protocol for geriatric emergency visits, including a preliminary assessment of GS.

## Data availability statement

The raw data supporting the conclusions of this article will be made available by the authors, without undue reservation.

## Author contributions

Y-TC: concept, design, acquisition, analysis, interpretation of data, and supervision. M-CL: drafting of the manuscript. C-CY and Y-TL: statistical analysis. F-DH: administrative, technical, or material support. All authors contributed to the article and approved the submitted version.

.
